# Co-Expression of Nogo-A in Dopaminergic Neurons of the Human Substantia Nigra Pars Compacta Is Reduced in Parkinson’s Disease

**DOI:** 10.3390/cells10123368

**Published:** 2021-11-30

**Authors:** Gian-Carlo Eyer, Stefano Di Santo, Ekkehard Hewer, Lukas Andereggen, Stefanie Seiler, Hans Rudolf Widmer

**Affiliations:** 1 Neurocenter and Program for Regenerative Neuroscience, Department of Neurosurgery, Inselspital, Bern University Hospital, University of Bern, 3010 Bern, Switzerland; gian-carlo.eyer@students.unibe.ch (G.-C.E.); stefano.disanto@insel.ch (S.D.S.); lukas.andereggen@ksa.ch (L.A.); stefanieseiler7@hotmail.com (S.S.); 2 Institute of Pathology, University of Bern, 3010 Bern, Switzerland; Ekkehard.Hewer@unil.ch; 3 Institute of Pathology, University of Lausanne, 1011 Lausanne, Switzerland; 4 Department of Neurosurgery, Kantonsspital Aarau, 5001 Aarau, Switzerland

**Keywords:** Nogo-A, Parkinson’s disease, tyrosine hydroxylase, substantia nigra pars compacta, human, immunofluorescence

## Abstract

Parkinson’s disease is mainly characterized by a progressive loss of dopaminergic neurons in the substantia nigra pars compacta. Together with the small number, the high vulnerability of the dopaminergic neurons is a major pathogenic culprit of Parkinson’s disease. Our previous findings of a higher survival of dopaminergic neurons in the substantia nigra co-expressing Nogo-A in an animal model of Parkinson’s disease suggested that Nogo-A may be associated with dopaminergic neurons resilience against Parkinson’s disease neurodegeneration. In the present study, we have addressed the expression of Nogo-A in the dopaminergic neurons in the substantia nigra in postmortem specimens of diseased and non-diseased subjects of different ages. For this purpose, in a collaborative effort we developed a tissue micro array (TMA) that allows for simultaneous staining of many samples in a single run. Interestingly, and in contrast to the observations gathered during normal aging and in the animal model of Parkinson’s disease, increasing age was significantly associated with a lower co-expression of Nogo-A in nigral dopaminergic neurons of patients with Parkinson’s disease. In sum, while Nogo-A expression in dopaminergic neurons is higher with increasing age, the opposite is the case in Parkinson’s disease. These observations suggest that Nogo-A might play a substantial role in the vulnerability of dopaminergic neurons in Parkinson’s disease.

## 1. Introduction

Parkinson’s Disease (PD) is the second most frequent neurodegenerative disorder, and among this category it is the fastest growing source of disability [1]. PD is age-related and typically diagnosed by the presence of motor deficits, including resting tremor, rigidity, bradykinesia and postural imbalance [2]. The primary trigger of the motor symptoms of PD consists in the depletion of dopamine in the striatum, resulting from a progressive loss of mesencephalic dopaminergic (DA) neurons projecting from the substantia nigra pars compacta (SNc). Currently there are no effective strategies to arrest the progression of PD [3].

The pathophysiology of PD is complex and relies on dysfunctions of different types of neuronal cells, circuits and structures of the central and autonomic nervous system. In this scenario, however, the death of nigral DA neurons is a crucial stage. There is evidence that DA neurons are particularly vulnerable [4]. Factors such as the extensive arborization and length of the axons, the relatively low cytosolic calcium buffering capacity and the potential toxicity of dopamine might predispose the DA neurons to dysfunctions. Endogenous and exogenous toxic insults, such as oxidative stress, accumulation of misfolded protein, inflammation and environmental toxins are the major causes of impairment of DA neuron viability. At the same time, it has been reported that subpopulations of DA neurons are more resilient to neurodegeneration [5,6,7]. Furthermore, a gradual and moderate decrease in DA neuron densities in the SNc appears to be a physiological process associated with aging, which seems to differ in some aspects to the neurodegenerative process occurring in PD [8,9,10,11]. These observations underscore the importance of a thorough comprehension of the determinants of DA neuron viability; a better characterization of DA neurons and in particular those spared during aging and in PD could help to unravel the mechanisms of neurodegeneration and identify new targets to modify its progression.

In our previous studies, we have hypothesized that the myelin-associated protein and potent inhibitor of neurite outgrowth Nogo-A (rericulon-4) might play a role in the DA neuron viability. Since the original description in the late 1980s, the array of functions of Nogo-A has widened [12]. It is now clear that different cell types in the neuronal tissue express Nogo-A besides oligodendrocytes, including neurons, endothelial cells, microglia [13,14]. Correspondingly, Nogo-A acts as modulator of neuronal circuit plasticity, angiogenesis, stem cell maturation and immune response [15,16]. Ourselves and others have also described that Nogo-A is expressed in cultured DA cells as well as in neurons in the SNc of adult rats [17,18,19]; a subpopulation of these cells were identified as projection neurons [12]. Importantly, we have previously reported an increased density of DA neurons co-expressing Nogo-A in an animal model of PD [18]. Compelling evidence indicates that Nogo-A signaling is involved in neuronal dysfunction and repair mechanisms in different neurodegenerative conditions, including spinal cord injury, ALS, Alzheimer’s and stroke [20,21,22,23] as well as psychiatric disorders such as schizophrenia [24]. Several studies have shown that the suppression of the Nogo-A-dependent inhibitory signals enhance neuronal plasticity and sprouting in damaged tissue as well as in neuronal transplantation settings. However, it is clear that a deep understanding of the changes occurring in damaged and aging neuronal tissue is essential to shape a promising therapeutic concept into safe and effective interventions [25]. In this context, the elucidation of Nogo-A expression might be relevant to address the pathogenesis of PD. However, the expression of Nogo-A in human SNc has not been reported yet. Hence, in the present study we aimed to characterize the expression of Nogo-A in DA neurons in the SNc obtained from PD and non-diseased subjects using a tissue micro array (TMA) platform. We have found a fairly abundant Nogo-A expression in human adult SNc and observed striking differences in the TH/Nogo-A co-localization rate in relation to age in the two populations. These results might advance our knowledge on the vulnerability of DA neurons.

## 2. Materials and Methods

### 2.1. Construction of the Human Substantia Nigra Pars Compacta TMA

**Figure 1:**
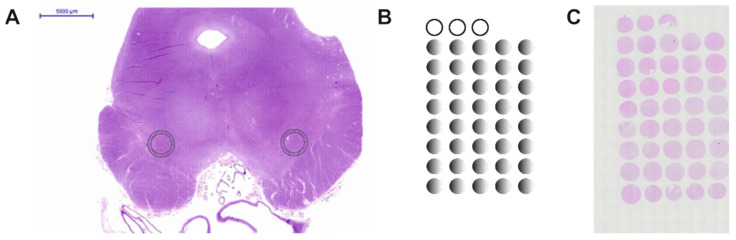
(**A**). Photomicrograph of a cross-section from a human mesencephalon stained with HE showing the area annotated for the punching of the SNc (circles). (**B**). Schematic drawing of a TMA with the human SNc samples (gray circles) and the non-neuronal tissue samples (open circles). (**C**). Representative photomicrograph of a TMA stained with HE.

**Figure A1:**
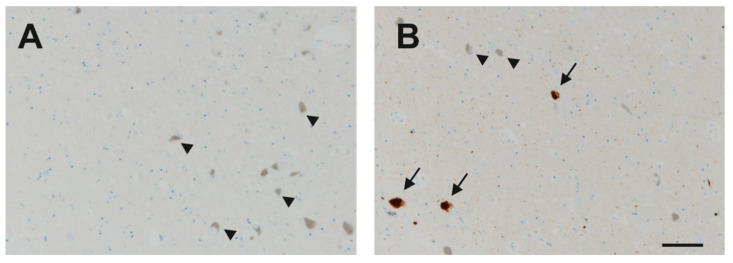
Microphotographs of the human SNc from a non-diseased sample (**A**) and from a PD specimen with α-synuclein (arrows) (**B**) inclusions. Note the pigmented DA neurons free of α synuclein (arrowheads). Scale bar: 100 µm.

The present study was performed with approval of the ethics committee of the Canton of Bern (KEK the 200/14). The TMAs were produced as described in detail previously [26,27]. In brief: stored human midbrain paraffin embedded tissue was obtained. Hematoxylin-eosin (HE) stained slides were scanned and photographed using a Pannoramic 250 (3DHistech). Ekkehard Hewer annotated areas in the left and right SNc suitable for the TMA (Figure 1A). These areas that were 2 mm in diameter were then punched out of the donor paraffin block and mounted onto a recipient block. TMA blocks presented with approximately 40 samples in which the first three samples included non-neuronal tissues, i.e., from kidney or liver for orientation of the array and control for the staining processes (Figure 1B). Non-diseased and PD samples were mounted in separate blocks with the samples sorted by the date of collection. The TMAs were sectioned at 2.5 µm thickness. The first slide was stained for HE and the second for α-synuclein. These stainings were performed to verify that all specimens in the TMAs were correctly annotated and in order to exclude that the non-diseased TMAs included brain tissues with α-synucleopathies (Appendix A, Figure A1).

A total of 56 cases were included for the non-diseased TMA (23 female and 33 male samples) for assessment of the age-dependent Nogo-A expression in dopaminergic neurons and analysis of Nogo-A-ir and tyrosine hydroxylase (TH) positive (TH-ir) cell densities. The mean age of all tissue samples was 67.1 ± 1.7 years, with similar ages for the female and male samples (67.5 ± 1.7 and 66.9 ± 2.0, respectively). The age of the samples extended from 32 to 91 years (females 32 to 91 years and males 41 to 88 years, respectively; Table A1 and Table A2).

For the diseased TMA samples of patients diagnosed with Morbus Parkinson (n = 19) were included, as shown in Table A3. The mean age of all tissue samples was 78.4 ± 1.2 years with similar ages for the female and male samples (77.3 ± 2.1 and 78.9 ± 1.5, respectively). The age of the samples ranged from 68 to 90 years (Table A3).

### 2.2. Immunohistochemistry

**Figure A2:**
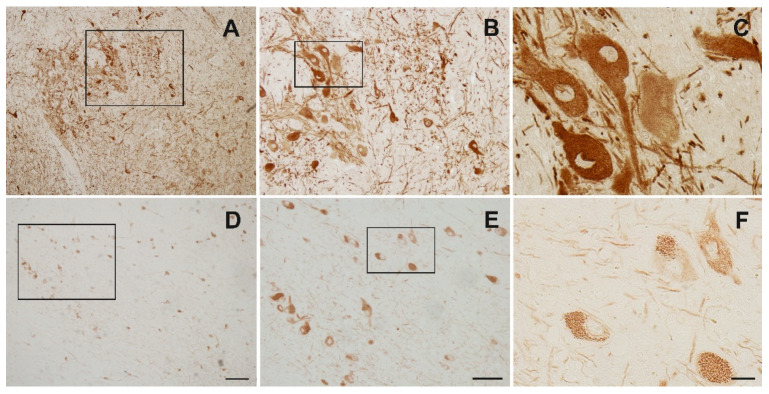
Representative digitalized photomicrographs of the human SNc stained for TH. Note the improved staining quality in the antigen retrieval treated sections (**A**–**C**) compared to not treated (**D**–**F**). (**B**,**E**) are magnifications from the box in (**A**,**D**), (**C**,**F**) are magnifications from (**B**,**E**). Scale bars: 200 µm (**A**,**D**), 100 µm (**B**,**E**), 20 µm (**C**,**F**).

Slides were dewaxed in two baths of fresh Xylol for 20 and 5 min, hydrated through descending ethanol concentrations (100%, 95%, 80%, 70% and 35% each for 3 min) and transferred to aqua dest. After two washes for 15 min with 0.1 M phosphate buffered saline (PBS) (pH 7.4), slides were exposed to epitope retrieval with citrate buffer (Appendix B; Figure A2). Thereafter, slides were blocked for 45 min with 10% horse serum in 0.1% Triton X-100 and incubated over night at 4°C with the primary antibodies (rabbit polyclonal anti-Nogo-A, Santa Cruz, sc-25660; mouse monoclonal anti-TH antibody, Millipore MAB5280; see also Appendix B) diluted in PBS containing 2.5% horse serum and 0.1% Triton X-100. Following washes in PBS (4 × 15 min) to remove unbound antibodies the sections were incubated for 2 h with secondary antibodies (Alexa-Fluor donkey anti-mouse 488 nm and Alexa-Fluor donkey anti-rabbit 594 nm or Alexa-Fluor donkey anti-mouse 594 nm and Alexa-Fluor donkey anti-rabbit 488 nm for the mouse and rabbit anti-Nogo-A antibodies, respectively; Thermo Fisher Scientific, Carlsbad, CA, USA, 1:250) and a fluorescent dye for detection of the cell nuclei (Hoechst 33352, 1:10000, Thermo Fisher Scientific). Thereafter the sections were washed in PBS (4 × 15 min) and mounted in 0.1 M PBS containing 50% glycerol.

### 2.3. Cell Count Analysis

Only cells with a clear morphology of neurons were counted in a blinded manner. Hence, Nogo-A positive cells that had a small cell body and looked morphologically similar to glia cells were not included. The number of cells were analyzed using an Olympus microscope (BX51) equipped with a motorized stage (MW Tango) that was connected to a digital camera (Olympus DP72) and connected to a PC with a calibrated neuron tracing software (Cellsens Dimension; Olympus Schweiz AG, Wallisellen, Switzerland). The following approach was used for the analysis of TH and Nogo-A positive neurons and the rate of co-localization: after acquiring multi-image alignments taken from each TMA, a virtual grid was overlaid and cells counted in the corresponding squares at a magnification of 10× (Appendix D; Figure A4). The number of TH positive, Nogo-A positive and cells expressing both markers per tissue sample was the sum of all fields analyzed covering this sample. The numbers were then transcribed into an excel spreadsheet Table and the co-localization rate was calculated. The specimens with a very weak or unclear TH-ir staining or complete absence of TH-ir cells were excluded from the TMA analysis. In 8 cases of the control TMA and 5 cases of the PD, only one side per specimen was available on the TMA, thus the counts were based on a single sample; otherwise, the mean value of the right and left SNc samples was taken.

### 2.4. Statistical Analysis

For statistical analysis, a commercially available software package was used (GraphPad Prism 7, La Jolla, CA, USA). Statistical significance of two groups only was assessed by two-tailed unpaired t-test or by the non-parametric Mann–Whitney test, based on the outcome of the D’Agostino and Pearson normality test. Linear regression was applied for correlation analyzes of co-localization rates and age. Statistical significance was set at *p* < 0.05. Data are presented as mean ± SEM.

## 3. Results

### 3.1. Nogo-A Is Expressed in DAneurons Human SNc

**Figure 2:**
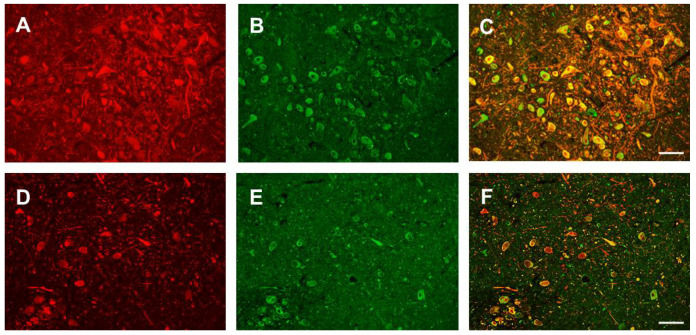
Digitalized photomicrographs of a 72-year-old PD male (upper row) and a 90-year-old PD male (lower row) specimen stained for tyrosine hydroxylase (**A**,**D**) and Nogo-A (**B**,**E**). Note the overall higher densities of neurons and a higher co-localization rate in the 72-year-old as compared to the 90-year-old specimen (**C**,**F**). Scale bars: 100 µm.

**Figure A3:**
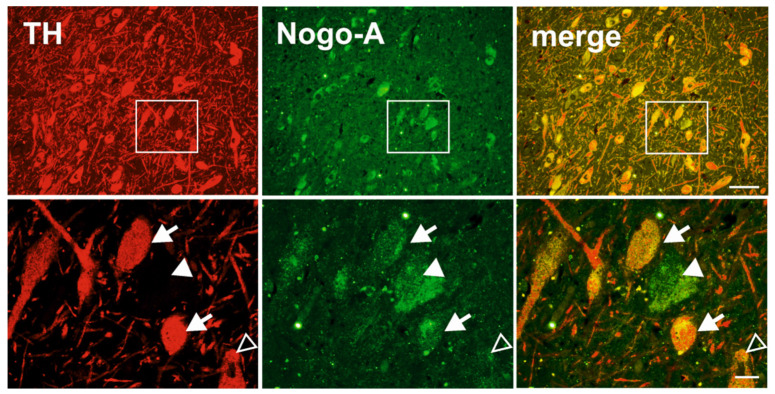
Representative digitalized photomicrographs of the human SNc stained for TH and Nogo-A. Lower panels show magnifications of boxed areas in the upper panels. Note some cells showed immunoreactivity for Nogo-A (arrowhead) or TH only (open arrowhead) while other neurons express both markers (arrows). Scale bars: 100 µm (top row), 20 µm (bottom row).

**Figure A4:**
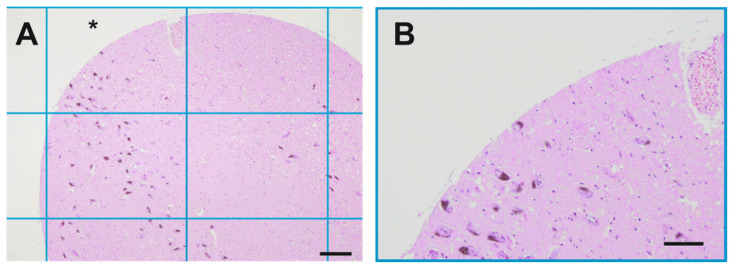
The photomicrograph shows a section of a HE stained MIA (**A**) taken at a magnification of 4× with an overlaid grid representative for the counting method. In each square (star) TH positive, Nogo-A positive cells and neurons expressing both markers were counted (magnification 10×; (**B**)). Scale bars: 200 µm (**A**), 100 µm (**B**).

We first determined that Nogo-A is expressed in human SNc. Immunohistochemical analysis using the DAB chromogen revealed a wide distribution and a specific staining in neuronal-like cells (Figure A2). We then verified that DA neurons express Nogo-A by means of a double immunofluorescence immunohistochemistry (Figure 2; Appendix C; Figure A3).

### 3.2. Co-Localization Rates Increase with Age in the Non-Diseased Brains

**Figure 3:**
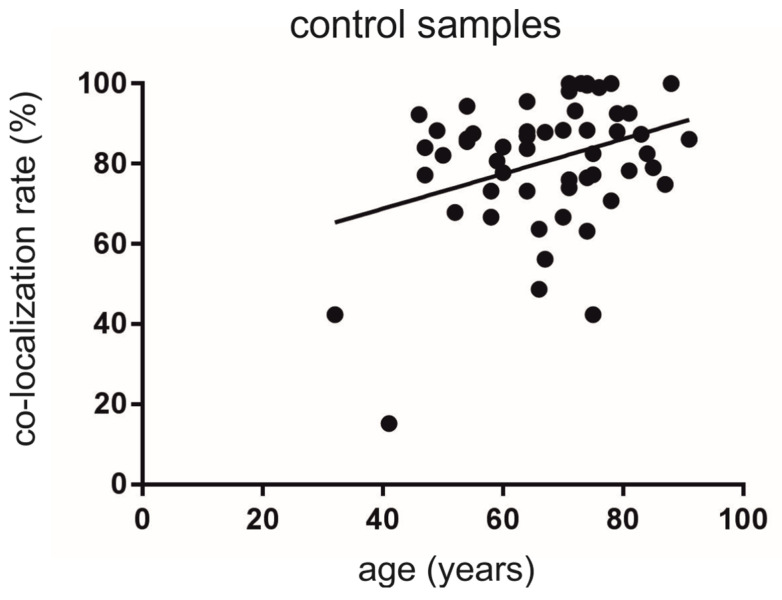
Analysis of the co-localization rates of TH-ir neurons also expressing Nogo-A depending on the age in the human SNc from non-diseased individuals. We found a significantly higher co-localization rate with increasing age (*p* < 0.05).

We detected a mean co-localization rate of 80.6 ± 2.2% for all analyzed TH-ir neurons expressing Nogo-A. Importantly, the co-localization rate increased with age and displayed a statistical significant correlation (Y = 0.4344 × X + 51.44; F (1, 54) = 6.878; r^2^ = 0.1130, *p* < 0.05) (Figure 3).

### 3.3. Lower Numbers of TH-ir Neurons in Normal Aging

**Figure 4:**
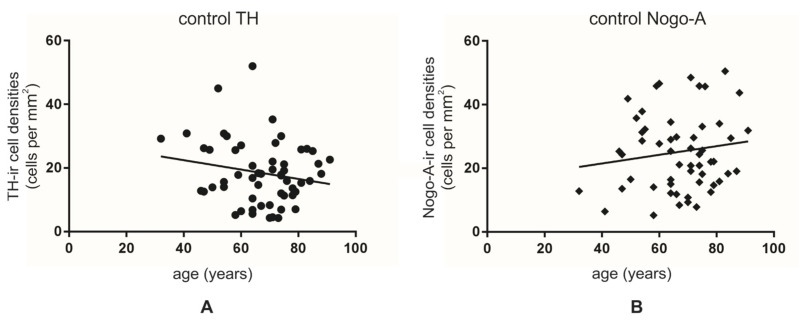
Analysis of TH-ir (**A**) and Nogo-A-ir cell densities (**B**) per mm^2^ depending on the age in the human SNc from non-diseased individuals. Both markers did not display a significant association with age.

**Figure A5:**
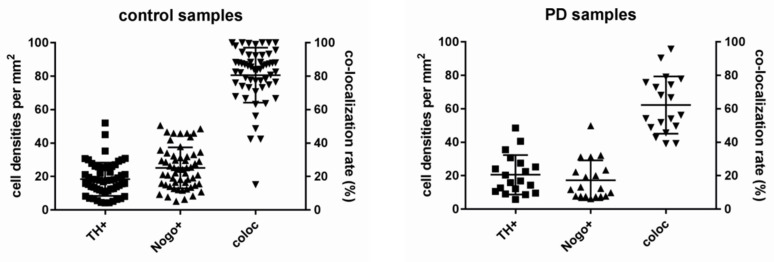
Summary of TH-ir, Nogo-A-ir cell densities per mm^2^ and rate of co-localization in control and PD samples. Values are given as mean ± SD.

The mean number of neurons was 18.5 ± 1.3 per mm^2^ and 25.2 ± 1.6 per mm^2^ for TH-ir and Nogo-A-ir neurons, respectively (Figure A5). When we assessed the number of TH-ir neurons depending on age, we detected a tendency for lower cell densities with increasing age (Y = −0.1474 × X + 28.35; F (1, 54) = 1.980; r^2^ = 0.0354, *p* = 0.165) (Figure 4A). No significant changes were observed for the density of Nogo-A-ir neurons (Y = 0.136 × X + 16.07; F (1, 54) = 1.103; r^2^ = 0.0200, *p* = 0.298) (Figure 4B).

### 3.4. Co-Localization Rates in PD Decrease Depending on Age

The mean co-localization rate of TH-ir neurons expressing Nogo-A was 62.3 ± 3.9% for all PD specimens (Figure A5).

**Figure 5:**
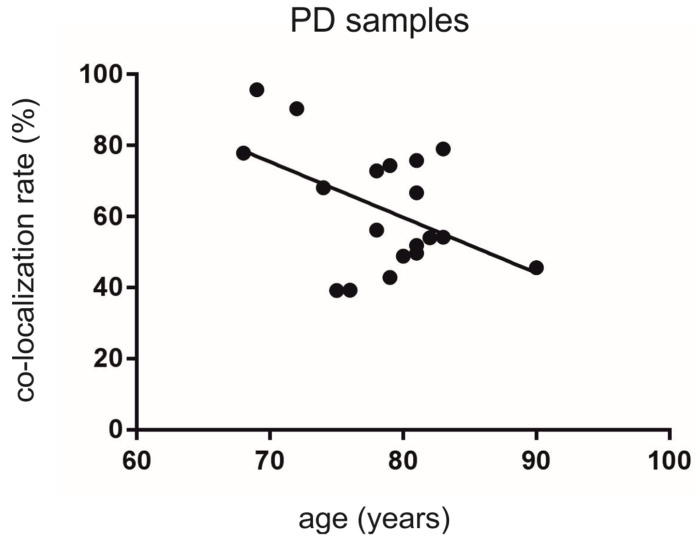
Analysis of the co-localization rates of TH-ir neurons also expressing Nogo-A depending on the age in the human SNc from PD individuals. There was a significant association with increasing age, showing lower levels of co-localization (*p* < 0.05).

Interestingly, we detected a significant decrease in the co-localization rate with increasing age for all PD samples included (Y = −1.562 × X + 184.8; F (1, 17 = 5.067; r^2^ = 0.2296, *p* < 0.05) (Figure 5).

### 3.5. Age Is Associated with Lower Numbers of TH-ir and Nogo-A-ir Neurons in PD

**Figure 6:**
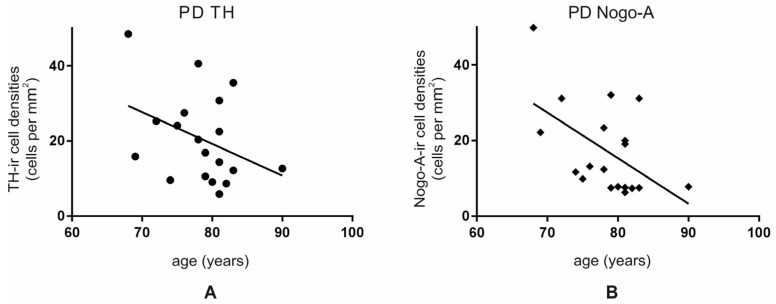
Analysis of TH-ir (**A**) and Nogo-A-ir cell densities (**B**) per mm^2^ depending on the age in the human SNc from PD individuals. Both markers displayed a significant decline with age.

The mean number of cells was 20.6 ± 2.7 per mm^2^ and 17.3 ± 2.7 per mm^2^ for TH-ir and Nogo-A-ir neurons, respectively. When we assessed the number of TH-ir and Nogo-A-ir neurons depending on age, we detected a decrease with increasing age. This decrease showed a tendency for TH-ir neurons (Y= −0.8993 × X + 91.52; F (1, 17) = 3.062; r^2^ = 0.1526, *p* = 0.1) (Figure 6A) and reached statistical significance for the number of Nogo-A-ir neurons (Y= −1.205 × X + 111.8; F (1, 17) = 6.661; r^2^ = 0.2815, *p* < 0.05) (Figure 6B).

## 4. Discussion

Aging is the primary risk factor of PD. Aging and PD share common features with aging, being considered a pre-parkinsonian state. In fact, although a general decline of nigro-striatal system functionality occurs during normal aging, this alone is not sufficient to cause PD. Rather, aging is associated with alterations at a cellular level that predispose the loss of DA neurons in the SNc, which is considered the cardinal event in the pathogenesis of PD [28]. Aging results in global changes in plasticity in the central nervous system, but SNc seems to be moderately affected compared to other brain regions [29]. Nogo-signaling plays a key role in the context of plasticity perturbations, and there is evidence that the expression of this complex signaling system is timely and regionally regulated in aging mice brains [30].

To the best of our knowledge, this is the first description of Nogo-A expression in human SNc in postmortem tissues from PD and non-diseased specimens. So far, Nogo-A in DA neurons has been reported in animals and in a human midbrain cell line [19,31]. Similar to previous observations in rodents, we report a fairly abundant Nogo-A co-localization with TH [18]. The principal finding of the present work consists of the substantial divergence of the TH/Nogo-A pattern of co-expression in relation with aging in PD compared to non-diseased specimens. In fact, TH-ir/Nogo-A-ir densities declined significantly with increasing age in the PD samples but not in the non-diseased. These differences are consistent with the concept of heterogeneity of DA neuron vulnerability [11] and seem to rely mostly on the Nogo-A-ir densities in the two groups. The TH-ir densities declining with age in the SNc are consistent with a progressive deterioration of the nigrostriatal system functionality during aging. Therefore, these results seem to support the hypothesis that consider PD as an exacerbation of the degenerative effects on the nigral DA neurons that accumulate during lifetime [32,33]. On the other hand, the opposite trends of TH/Nogo-A co-expression during aging in the two experimental groups examined suggest that distinct phenotypic changes in the SNc landmark the pathogenesis of PD. Accordingly, past investigations on the ultrastructural abnormalities in dying DA neurons have disclosed that the apoptotic processes differ between successful aging and Parkinson’s disease [8,28]. The ascending slope of Nogo-A expression with age in the non-diseased SNc samples is another striking observation of our study. These results challenge the current knowledge on Nogo-A expression in the aging brain. In fact, it has been reported that in mouse brains, the expression of Nogo-A protein declines during aging [34]. Others have associated the reduced levels of Nogo-A mRNA in the cortex and hippocampus of aging rats with a decline in synaptic plasticity [35]. Based on the quantitative gene expression level analysis in different brain areas (but not including the mesencephalon), Smedfords and colleagues concluded that in adulthood and aging the Nogo signaling system is extremely stable [30]. We have previously found that one month after intrastriatal lesion with 6-OHDA, rats show a substantial loss of Nogo-A expressing cells in the SNc that parallel the reduction in TH-ir neurons [18]. However, we have also reported that TH-ir neurons’ vulnerability to 6-OHDA differs according to the co-expression of Nogo-A, with the Nogo-A-ir neurons better withstanding the toxic insult [18]. The results of the present study hence represent a shift in the paradigm of Nogo-A/TH co-localization in respect to our earlier findings. These results raise fundamental questions; namely, whether the Nogo-A expression identifies a population of nigral neurons with different vulnerability and functions or the alterations in Nogo-A expression pattern are the cause/effect of the changes occurring in normal aging and PD samples. It is intriguing to speculate that an increase in Nogo-A expression in DA neurons underlies compensatory adaptations to cope with mild or acute degenerative conditions, such as those occurring in aging in humans or those exerted by 6-OHDA in rats, respectively. There is growing evidence that Nogo participates in different processes related to apoptosis and autophagy [36,37]. However, whether Nogo-A plays a direct role in regulating the dopaminergic neurons viability in healthy and diseased brains is currently not known. These features of Nogo-A offer perspectives for novel interventions to contrast cognitive decline in aging and pathogenic neurodegeneration. Indeed, suppression of Nogo-A signaling supports functional regeneration by promoting neuronal plasticity and axonal sprouting. Moreover, a number of preclinical and clinical studies have demonstrated that Nogo-A inhibition has a therapeutic potential for neurodegenerative conditions, such as PD [16], MS [38], spinal cord injury [39] and stroke [40].

The TMA methodology employed in the present work is a powerful tool for exploring new biomarkers and drug targets for neurodegenerative diseases such as PD. Few general and specific caveats of this technology should also be acknowledged. These limitations are inherent to the TMA technique [41]. With regards to the present work, it was not possible to retrieve information about underlying diseases in the samples as well as the agonal state and postmortem interval. Moreover, despite the efforts for an accurate sampling methodology, small variations in the sampling region cannot be excluded. In fact, besides the individual neuroanatomical differences, it should be considered that the core size on the array is considerably small when compared to the entire SNc from which it has been isolated. Furthermore, the analyses (counting) were not automated. However, even considering these limitations, the TH/Nogo-A co-localization displays a clear association with age in the non-diseased as in the PD cohort. In the context of variability, we have observed different co-localization and cell density rates among the right and left SNc samples of the same specimen. These differences might be the result of the sampling methodology, but might also undermine the nature of the physiology of neurodegeneration, which has typically a unilateral onset in PD [3]. Clearly, the lack of clinical information of the sample donors, including the stage of the disease, time of disease onset and types of symptoms (including motor and no-motor symptoms) limit the possibility to infer detailed conclusions regarding the differences of TH/Nogo-A co-localization in SNc in the context of PD neurodegeneration.

Together with age, sex is an important risk factor of PD, with women being less prone to develop the disease [42,43] and with a delayed onset of the motor symptoms compared to men [44]. Moreover, non-motor symptoms are more severe and frequent in women [45], while the cognitive decline progresses more rapidly in male PD patients [46]. The reasons underlying this difference are not completely understood but multiple observations suggest that estrogen has neuroprotective effect on the nigrostriatal dopaminergic system [47]. Unfortunately, due to the small number of samples, the present study does not allow us to define whether differences in TH/Nogo-A co-expression might contribute to sex differences in dopaminergic neuron vulnerability, as recently described for other markers such as VGLUT [48].

The present study highlights this perspective by dissecting the biology of Nogo-A. Understanding the function of Nogo-A in DA neurons might shed light on the pathogenesis of PD and eventually open new scenarios for its diagnosis and therapy. For this purpose, further analyses in larger groups of samples are needed. In particular, the relation with the several factors involved in the onset and progression of the disease, including the differences in aging females and males, the effect of the severity of the symptoms and the association with other markers of neurodegeneration as α-synuclein need to be addressed in more detail.

## 5. Conclusions

The relevance of the changes in the TH/Nogo-A expression pattern reported here remains to be investigated, but to the best of our knowledge, this study is the first to characterize the alterations in these markers’ co-expression in human postmortem tissue samples. These results might advance our understanding of the vulnerability of DA neurons. The examination of TH/Nogo-A co-localization might thus provide a novel perspective to understand the spectrum of alterations occurring in PD and develop novel therapeutic approaches.

## Data Availability

Data are contained within the article.

## Appendix A. Immunohistochemistry for α-Synuclein

The stainings for α-synuclein were performed by the Institute of Pathology, University of Bern. The TMA slides were dried and baked at 60 °C for 30 min prior to use. Immunostaining was performed by automated staining using Bond RX (Leica Biosystems) immunostainer using the following protocol: Slides were dewaxed in Bond dewax solution (product code AR9222, Leica Biosystems). Heat-induced epitope retrieval (EDTA based pH 9.0; code AR9640, Leica Biosystems) was performed for 30 min at 95°. Mouse anti α-synuclein antibodies (Novocastra NCL-L-ASYN) were diluted 1:200 and incubated for 30 min. Samples were incubated with horseradish peroxidase-polymer for 15 min and subsequently visualized using 3,3-diaminobenzidine (DAB) as brown chromogen (Bond polymer refine detection, Leica Biosystems, Ref DS9800) for 10 min (Figure A1).

**Table A1:** Female specimens included in the non-diseased TMA.

Sample Number	Age
1	32
2	46
3	50
4	52
5	55
6	58
7	59
8	64
9	64
10	64
11	67
12	70
13	73
14	74
15	74
16	74
17	75
18	79
19	79
20	83
21	84
22	85
23	91

**Table A2:** Male specimens included in the non-diseased TMA.

Sample Number	Age
1	41
2	47
3	47
4	49
5	54
6	54
7	54
8	58
9	60
10	60
11	64
12	64
13	64
14	66
15	66
16	67
17	70
18	71
19	71
20	71
21	71
22	72
23	74
24	74
25	75
26	75
27	76
28	78
29	78
30	81
31	81
32	87
33	88

**Table A3:** Specimens included in the PD TMA.

Sample Number	Gender	Age
1	Female	69
2	Female	75
3	Female	76
4	Female	80
5	Female	81
6	Female	83
7	Male	68
8	Male	72
9	Male	74
10	Male	78
11	Male	78
12	Male	79
13	Male	79
14	Male	81
15	Male	81
16	Male	81
17	Male	82
18	Male	83
19	Male	90

## Appendix B. Immunohistochemistry for Establishment of Best Staining Method

Slides were first dewaxed in fresh Xylol for 20 min and another 5 min, hydrated through descending ethanol concentrations (100%, 95%, 80%, 70%, 35% each for 3 min) and transferred to aqua dest. After two washes for 15 min with 0.1 M phosphate buffered saline (PBS) (pH 7.4) slides were exposed to epitope retrieval. This was carried out by boiling the sections in citrate buffer (10 mM Citric acid, 0.05% Tween 20, pH 6.0) for 30 min. Slides were cooled down for 20 min and washed in 0.1 M PBS for 10 min prior to the immunhistochemical stainings (vide infra). Unmasking antigens after fixation resulted in an enhanced the staining intensity for TH (Figure A2).

Thereafter, slides were blocked for 45 min with 10% horse serum in 0.1% Triton X-100 and incubated over night at 4 °C with the primary antibodies diluted in PBS containing 2.5% horse serum and 0.1% Triton X-100. Following washes in PBS (4 × 15 min) to remove unbound antibodies, the sections were incubated for 2 h with biotinylated secondary antibodies (Vector Laboratories) diluted 1:200 in PBS containing 0.1% Triton X-100 and 2.5% horse serum. The endogenous peroxidase activity was blocked with a solution of 10% H_2_O_2_ and 10% methanol in 0.1 M PBS for 10 min. Thereafter, the sections were washed in PBS (4 × 15 min) and subsequently incubated for 60 min at room temperature with an avidin-biotin-peroxidase complex (VECTASTAIN^®^ ABC-Peroxidase Kit; 1:250, PK-4000; Vector Laboratories, Servion, Switzerland) in combination with the 3,3-diaminobenzidine (DAB) Substrate Kit (34002, ThermoFischer Scientific). Finally, the sections were dehydrated in a series of alcohol dilutions (70%, 95%, 100%, alcohol), cleared in Xylene and mounted in Eukitt.

In order to find the best staining protocol for detection of Nogo-A and TH in the TMAs, we used sections of a 45-year-old non-parkinsonian male specimen. Both the rabbit polyclonal anti-Nogo-A antibody (Santa Cruz, sc-25660) in combination with the mouse monoclonal anti-TH antibody (Millipore, MAB5280) were used at different dilutions. The polyclonal Nogo-A antibody has been used successfully by our and other labs performing immunohistochemical analyses in rat, mouse and monkey tissues [18,19,49,50,51,52]. Similarly, the anti-TH antibody has been shown to be a specific marker for TH in our lab for many years [16,18,53,54].

Based on the outcomes of our extensive test stainings, we performed the quantifications of the TMA’s using the anti-Nogo-A and the anti-TH antibody at a dilution of 1:250 and 1:1000, respectively and antigen retrieval.

## Appendix C. A Subpopulation of Neurons in the SNc Express Nogo-A Only

Subpopulations of neurons only expressed one marker, i.e., TH or Nogo-A (Figure A3). Overall, about 20% of the Nogo-A positive neurons did not co-localize with TH while 80% did. Hence, more Nogo-A neurons presented with co-localization.

## Appendix D. Cell Count Approach

On the acquired multi-images taken from each TMA, a virtual grid was overlaid and cells counted in the corresponding squares at a magnification of 10× (Figure A4). The area under the 10× magnification was 577,284 μm^2^. Hence, cell numbers counted in all fields of a specimen were multiplied by a factor of 1.732, so we could extrapolate for the TH-positive and Nogo-A positive cell densities per mm^2^. The number of cells was the sum of all fields analyzed covering this sample. A summary of the gathered data is given in Figure A5.
